# Same species, different prerequisites: investigating body condition and foraging success in young reef sharks between an atoll and an island system

**DOI:** 10.1038/s41598-019-49761-2

**Published:** 2019-09-17

**Authors:** Ornella C. Weideli, Ian A. Bouyoucos, Yannis P. Papastamatiou, Gauthier Mescam, Jodie L. Rummer, Serge Planes

**Affiliations:** 1 PSL Research University: EPHE-UPVD-CNRS, USR 3278 CRIOBE, 66860 Perpignan, France; 2 SOSF - D’Arros Research Centre (SOSF-DRC), c/o Save Our Seas Foundation (SOSF), CH-1201 Geneva, Switzerland; 3 Australian Research Council Centre of Excellence for Coral Reef Studies, James Cook University, Townsville, Queensland 4811 Australia; 4 Department of Biological Sciences, Marine Sciences Program, Florida International University, North Miami, Florida 33181 USA; 5 Projects Abroad, Shark Conservation Project Fiji, West Sussex, BN124TX United Kingdom; 6 Laboratorie d’Excellence ‘CORAIL’, EPHE, PSL Research University, UPVD, CNRS, USR 3278 CRIOBE, Papetoai, Moorea, French Polynesia

**Keywords:** Community ecology, Behavioural ecology

## Abstract

Acquiring and storing energy is vital to sharks of all age-classes. Viviparous shark embryos receive endogenous maternal energy reserves to sustain the first weeks after birth. Then, in order to maintain body condition, sharks must start foraging. Our goal was to understand whether maternal energy investments vary between blacktip reef sharks (*Carcharhinus melanopterus*) from two populations and to what extent body condition and the initiation of foraging might be affected by presumably variable maternal investments. A total of 546 young sharks were captured at St. Joseph atoll (Seychelles) and Moorea (French Polynesia) between 2014 and 2018, and indices of body condition and percentage of stomachs containing prey were measured. Maternal investment was found to be site-specific, with significantly larger, heavier, and better conditioned individuals in Moorea. Despite these advantages, as time progressed, Moorea sharks exhibited significant decreases in body condition and were slower to initiate foraging. We suggest that the young sharks’ foraging success is independent of the quality of maternal energy resources, and that other factors, such as prey availability, prey quality, and/or anthropogenic stressors are likely responsible for the observed differences across sites. Insights into intraspecific variations in early life-stages may further support site-specific management strategies for young sharks from nearshore habitats.

## Introduction

Acquiring and storing energy reserves to maintain body functions and survival is vital to animals of all age-classes^1^. To estimate energy reserves during various life-stages, body condition, as a proxy of animal health, is commonly used^2^, with animals in good body condition presumably associated with relatively larger energy reserves^2,3^. At birth, an animal’s body condition is determined by the parents, notably by the mother^4^. Depending on maternal size and age at parturition, the diet, as well as the environmental conditions to which the mother was exposed during gestation, the offspring’s size, body mass, and body condition can vary among and within species. Indeed, coral reef fishes from high quality habitats pass on larger yolk reserves to their offspring than parents living in low quality habitats^5^. In the first weeks after birth, young animals with no parental care are required to gradually incorporate autonomous foraging activities to their daily routine to sustain their energy reserves. Hence, young animals depend on prey resources and habitat quality, in addition to remaining maternal energy resources. While strong positive relationships between parental energy reserves and factors such as offspring condition and time to exogenous feeding have been noted for teleost fishes and marine reptiles^5–8^, little work has been done on maternal energy investment in elasmobranchs. As maternal investment may vary with life-history traits (e.g., size, body condition) and habitat, it is also important to understand if and to what extent the level of maternal energy investment affects the offspring’s condition and foraging development during the first weeks of life.

Elasmobranchs occur across a range of heterogenous habitats and experience variable environmental conditions and levels of anthropogenic threats that differentially affect life-history traits^9^. While intraspecific differences in life-history traits may be less distinctive in sharks with broad movement patterns, genetically and geographically isolated sharks with restricted movements and site-fidelity are known to exhibit pronounced intraspecific differences in size at birth, growth rates, and litter sizes^9,10^. Adult reef-sharks from the family Carcharhinidae have been the focus of a number of studies investigating such differences^10^, but fewer studies have characterized intra-specific variability among populations of young animals. Barker *et al*.^11^, for example, reported that larger sized female lemon sharks (*Negaprion brevirostris*) from Florida’s Marquesas Keys (USA), give birth to larger offspring than the smaller sized females from a nearby nursery^11^. Likewise, Hussey *et al*.^12^ revealed an increase in maternal reproductive output (larger neonatal mass) with increasing maternal size in two carcharhinid sharks. While such size and body mass measurements can help assess characteristics of young shark populations^13^, their relationship (e.g., body mass per unit size) provides invaluable information on energy reserves, overall body condition and fitness^2,3,14,15^. Despite the importance of energy reserves, it is unclear as to whether maternal energy investment varies across shark populations adopting different life-history traits.

At birth, viviparous sharks receive endogenous energy reserves, primarily stored as lipids in livers, from their mothers^12,16–18^. Although this maternal energy allocation can lead to significantly enlarged livers in neonatal sharks (up to 20% of total body mass^12^), this endogenous energy transfer is finite. As opposed to marine mammals, where exogenous maternal energy provisioning (e.g., lactation) can last months to years, depending on species^19^, sharks receive no maternal aftercare. This results in energy resources being utilized within the first weeks, as demonstrated by decreasing condition indices^12,17^. Similarly, Duncan and Holland^16^ reported mass loss in young sharks following parturition, most likely a sign of depleted energy reserves. To counteract such declines, young sharks are required to incorporate autonomous foraging to their daily routine. As it is difficult to directly observe young sharks foraging in the wild, biomarkers that indirectly estimate when young sharks shift from relying on maternal energy resources to feeding autonomously have recently been established^20,21^. Although biomarkers, such as bulk stable isotopes, alone^20,22^ or in combination with fatty acids^21^, provide insights into autonomous foraging developments and broad estimates of body condition^23,24^, isotopic turnover rates impede timely and precise estimates of body condition as well as foraging development. To date, Hussey *et al*.^12^ executed the only study that simultaneously and precisely assessed changes in body condition and estimated foraging development in early life-stages of sharks^12^ via a combination of lethal and non-lethal measurements to calculate such developments across different umbilical scar healing stages. Given that the study only focused on a single location that precludes investigations on variabilities among populations, further work is needed to understand if and how body condition and the development of autonomous foraging may vary across species inhabiting different habitats.

To examine intraspecific variability in body condition and foraging development during the first weeks of life, we collected life-history data from neonatal and juvenile blacktip reef sharks (*Carcharhinus melanopterus*), a species with high levels of genetic population structure^25,26^, from two remote habitats in the Indo-Pacific Ocean. While Moorea (French Polynesia) is a remote island with human-impacted shorelines in the South Pacific, St. Joseph atoll (Seychelles), located in the western Indian Ocean, consists of a small and uninhabited ring of islands with adjacent shallow reef flats. Our objectives were to use non-lethal methods to determine (1) whether maternal energy investment varies between *C. melanopterus* populations potentially adopting different life-history strategies, and (2) if and to what extent body condition and foraging development might be affected by presumably variable maternal investments. We hypothesized that better conditioned neonates (e.g., neonates with higher energy stores) would show a slower decrease in body condition and a faster acquisition of foraging skills during the first weeks of life. Considering the steady increase in human activities in nearshore areas that is resulting in declining prey abundance and habitats^16,27,28^ as well as predicted higher water temperatures due to climate change^29,30^, a better understanding of maternal energy investments, body condition, and autonomous foraging development during early life-stages of sharks has important implications for conservation^31^. Insights into potential intraspecific differences in such characteristics may further support site-specific management strategies for sharks from remote and potentially prey-limited habitats^32,33^.

## Results

### Intraspecific variation in maternal energy investments

**Figure 1:**
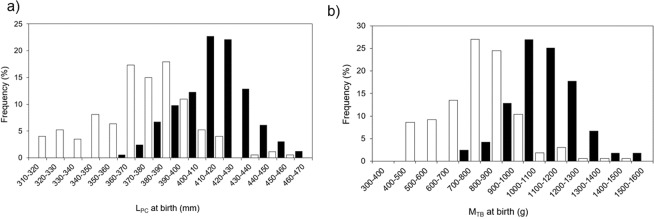
Percentage frequency histogram of (**a**) precaudal length (L_PC_) and (**b**) total body mass (M_TB_) in neonatal *Carcharhinus melanopterus* (USS1 and USS2) from Moorea (black, n = 163) and St. Joseph (white, n = 173, 164 respectively).

In Moorea, during the parturition seasons in 2016/2017 and 2017/2018, a total of 313 neonatal and juvenile *C. melanopterus* were captured and measured. Of those, 163 individuals (52%) were categorized as neonates (based on the presence of open or semi-healed umbilical scars) ranging from 368 to 466 mm L_PC_ (418.42 ± 18.90 mm, Fig. 1a) and weighting 670 to 1500 g (1025.22 ± 148.75 g, Fig. 1b). At St. Joseph, during the parturition seasons in 2014/2015, 2015/2016 and 2016/2017, a total of 233 neonatal and juvenile *C. melanopterus* were collected. Of those, 173 individuals (74%) were categorized as neonates ranging from 287 to 459 mm L_PC_ (372.22 ± 27.66 mm, Fig. 1a) and weighting 300 to 1375 g (694.99 ± 182.71 g, Fig. 1b). Neonatal *C. melanopterus* from Moorea were significantly larger (two sample *t*-test: t = 17.769, df = 334, p < 0.0001) and heavier at birth (two sample *t*-test: t = 17.917, df = 325, p < 0.0001) than individuals from St. Joseph. Mean water temperatures during the pupping seasons were significantly lower in Moorea (29.5 °C ± 0.003) compared to St. Joseph (30.0 °C ± 0.003; two sample *t*-test: t = −101.87, df = 1040400, p < 0.0001; see Supplementary Information S1).

**Figure 2:**
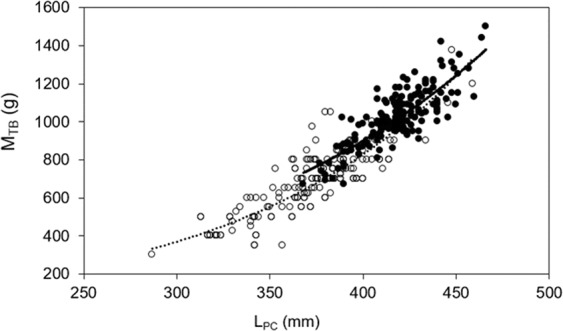
Relationship between total body mass (M_TB_) and precaudal length (L_PC_) of neonatal *Carcharhinus melanopterus* (USS1 and USS2) from Moorea (black, y = 0.042472x^2.70^, r^2^ = 0.69, n = 163) and St. Joseph (y = 0.013947x^2.98^, r^2^ = 0.73, n = 164).

**Figure 3:**
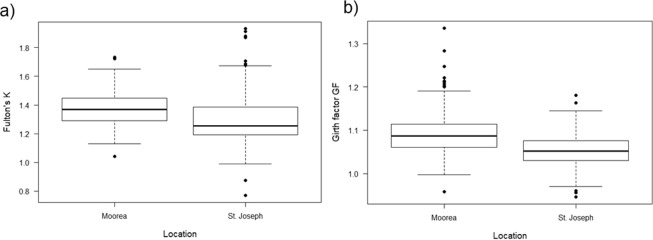
Comparison of body condition indices across locations. (**a**) Fulton’s K for neonatal and juvenile *Carcharhinus melanopterus* from Moorea (n = 313) and St. Joseph (n = 224). (**b**) Girth factor GF for neonatal and juvenile *Carcharhinus melanopterus* from Moorea (n = 313) and St. Joseph (n = 91). Boxes indicate the interquartile range with the median shown by horizontal lines, minimum and maximum values shown by whiskers, and points representing outliers.

Body condition, as calculated via three methods, differed significantly across locations. Neonatal sharks from Moorea were heavier for any given size than those at St. Joseph (F_1,234_ = 20.89, p < 0.001), and length-body mass results suggest positive allometric growth (Fig. 2). Independent indices of body condition were significantly higher in Moorea sharks compared to St. Joseph sharks, as calculated by Fulton’s K (two sample *t*-test: t = 6.083, df = 535, p < 0.0001; Fig. 3a) and GF (two sample *t*-test: t = 7.036, df = 402, p < 0.0001; Fig. 3b). Linear regressions revealed no relationships between L_PC_ and Fulton’s K (*St. Joseph*: F_1,222_ = 2.247, p = 0.135), and L_PC_ and GF (*Moorea*: F_1,311_ = 1.035, p = 0.310; *St. Joseph*: F_1,89_ = 3.397, p = 0.069; see Supplementary Information S2). However, linear regressions revealed significant negative relationships between L_PC_ and Fulton’s K in Moorea sharks (F_1,311_ = 11.280, r^2^ = 0.04, p = 0.0009; see Supplementary Information S2).

### Intraspecific variation in change of body condition

**Figure 4:**
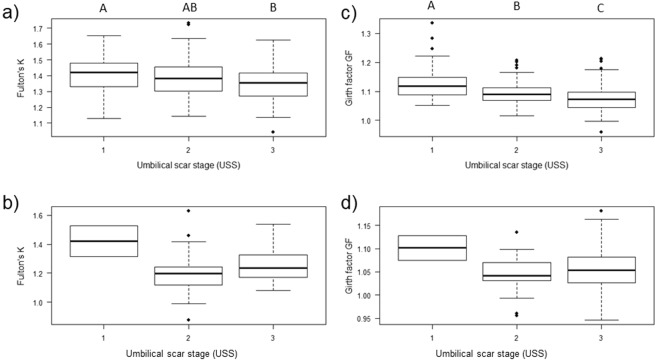
Transition of body condition indices with increasing umbilical scar stages (USS) in *Carcharhinus melanopterus*. Fulton’s K at (**a**) Moorea and (**b**) St. Joseph, and girth factor GF at (**c**) Moorea, and (**d**) St. Joseph. Boxes indicate the interquartile range with the median shown by horizontal lines, minimum and maximum values shown by whiskers, and black dots represent outliers. Letters above plots in (**a**) and (**c**) indicate statistically significant differences between groups. Sample size in Moorea: USS1 n = 59, USS2 n = 104, USS 3 n = 150. Sample size at St. Joseph: USS1 n = 2, USS2 n = 29, USS3 n = 60.

In cases where body condition indices did not conform to a normal distribution (Shapiro-Wilks test, p < 0.05), non-parametric one-way and multiple comparison tests were applied. Body condition, as estimated by Fulton’s K in young sharks from Moorea, differed significantly among increasing umbilical scars stages (ANOVA, F_2,310_ = 6.907, p = 0.001). Specifically, pair-wise comparisons showed statistical differences between USS1 and USS3 (Tukey’s HSD, *t* = −0.06, p = 0.001) and decreasing, albeit non-significant, body condition between USS1 and USS2 (Tukey’s HSD, *t* = −0.03, p = 0.179) and between USS2 and USS3 (Tukey’s HSD, *t* = −0.03, p = 0.097, Fig. 4a). Body condition, as estimated by girth factor GF in young sharks from Moorea, decreased significantly as umbilical scar stages increased (Kruskal-Wallis test, χ^2^ = 48.513, df = 2, p < 0.001). Pair-wise comparisons reported significant differences between all three umbilical scar stage classes (Dunn test, USS1/USS2: p = 0.0006; USS1/USS3: p < 0.0001; USS2/USS3: p = 0.0003, Fig. 4c). On the contrary, no significant differences were found between Fulton’s K and GF with increasing umbilical scar healing stages in young sharks from St. Joseph (Kruskal-Wallis test, χ^2^ = 8.6627, df = 2, p = 0.056 and χ^2^ = 2.8051, df = 2, p = 0.246, respectively; Fig. 4b,d).

**Figure 5:**
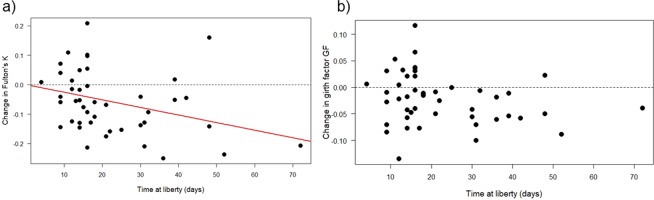
Changes in body condition indices with time at liberty in *Carcharhinus melanopterus* from Moorea. (**a**) Change in Fulton’s K over time at liberty, and (**b**) change in girth factor GF over time at liberty. Data were obtained from neonatal sharks that were measured twice within the same parturition season (min. 4 days, max. 72 days, n = 45). The regression line for predicting changes in Fulton’s K from time at liberty is shown in red (y = 0.001–0.003x, r^2^ = 0.11). Note that each dot represents the change of body condition in one individual and that negative values (below the dashed line) depict a decrease of body condition in an individual shark.

**Figure 6:**
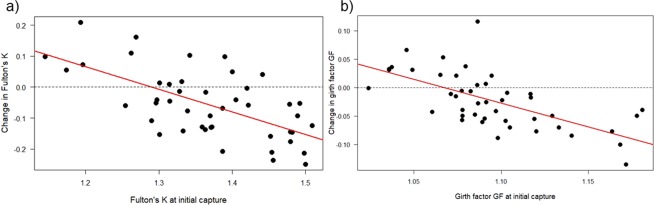
Changes in body condition indices versus body condition indices at initial capture in neonatal *Carcharhinus melanopterus* from Moorea. (**a**) Change in Fulton’s K versus Fulton’s K at initial capture, and (**b**) change in girth factor GF versus girth factor GF at initial capture. Data were obtained from sharks that were measured twice within the same parturition season (min. 4 days, max. 72 days, n = 45). The regression lines are shown in red (K: y = 0.94 - 0.73x, r^2^ = 0.40 and GF: y = 0.90 - 0.84x, r^2^ = 0.42, respectively). Note that each dot represents the change of body condition in one individual and that negative values (below the dashed line) depict a decrease of body condition in an individual shark.

The 45 individuals that were recaptured in Moorea during one parturition season were at liberty from 4 to 72 days (see Supplementary Information S3), and linear regressions showed significant negative relationships between changes in Fulton’s K and time at liberty (F_1,43_ = 5.41, p = 0.025, r^2^ = 0.11, Fig. 5a). Linear regression further revealed decreasing, albeit non-significant, relationships between GF and time at liberty (F_1,45_ = 2.75, p = 0.104; Fig. 5b). Similarly, linear regression indicated significant negative relationships between change in body condition with body condition at initial capture (Fulton’s K; F_1,43_ = 28.46, r^2^ = 0.40, p < 0.0001; Fig. 6a; GF: F_1,43_ = 31.71, r^2^ = 0.42, p < 0.0001, Fig. 6b). When differences in body condition indices were regressed against one another, data showed that changes in Fulton’s K could be predicted by changes in GF (F_1,43_ = 16.83, r^2^ = 0.28, p = 0.0002; see Supplementary Information S4), suggesting that estimates of either condition index were consistent within individuals.

### Intraspecific variation in foraging success

**Figure 7:**
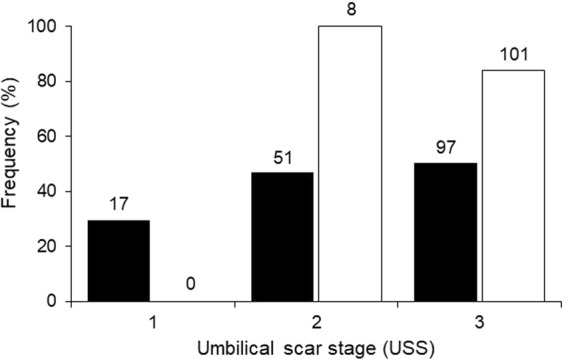
Frequency histogram of percentage stomachs containing prey with increasing umbilical scar stage (USS) in *Carcharhinus melanopterus* from Moorea (black, n = 165) and St. Joseph (white, n = 109). Numbers above each column represent the total sample size of sharks for a given umbilical scar stage (USS) on which gastric lavages were performed.

In Moorea, over the scope of one parturition season (year 2016/2017), 165 gastric lavages in *C. melanopterus* resulted in 78 full (47%), and 87 empty (53%) stomachs. At St. Joseph, over the scope of two parturition seasons (years 2015/2016 and 2016/2017), 109 gastric lavages in young *C. melanopterus* provided 93 full (85%) and 16 empty (15%) stomachs, leading to a significant bias of stomach fullness with locations (Χ_2_ = 36.60, p < 0.0001). When separated by USS, the frequency of stomachs containing prey items (increased foraging success) increased from 30% (USS1; n = 17) and 47% (USS2; n = 51) to 51% by USS3 in Moorea (n = 97; Fig. 7). At St. Joseph at USS2, 100% of sampled stomachs had prey items in them (n = 8), and 84% of 101 individuals at USS3 had stomachs containing prey (Fig. 7). The smallest acrylic tubes (2.5 cm outer diameter) were still too large to be used with the smallest individuals from St. Joseph, resulting in a lack of sampled USS1 individuals.

## Discussion

This study represents the first non-lethal investigation of body condition and concurrent estimates of autonomous foraging development in young *C. melanopterus* from two isolated shark populations. Our data provide compelling evidence that maternal investment is site-specific, with significantly larger sizes, greater body masses, and larger body condition measurements in Moorea sharks when compared to St. Joseph sharks. Furthermore, our data suggest that, despite this better head start, young sharks in Moorea exhibited significant decreases in body condition and developed foraging habits slower than sharks from St. Joseph (e.g., fewer than half of the stomachs lavaged from sharks in Moorea had contents during later development stages). These differences in foraging success likely explain the significant decrease in body condition in Moorea sharks, while sharks at St. Joseph maintained their body condition. Likewise, data from recaptured individuals from Moorea confirm the significant decrease in body condition with increasing time at liberty (up to 72 days). Recaptured individuals that initially had higher body condition indices were most likely to exhibit declines in body condition during the first weeks/months of life.

The fact that we observed larger and heavier neonates with greater mass per unit length and higher condition indices in Moorea versus St. Joseph suggests that neonates in Moorea are being well provisioned by larger, better conditioned mothers with potentially lower fecundity. Indeed, adult *C. melanopterus* from Moorea tend to be larger^34^ than adult *C. melanopterus* from St. Joseph^35^ and are therefore likely to produce larger, heavier and better conditioned young^12^. Body size is strongly heritable, and it’s also common for geographically separated shark populations of the same species to be genetically and morphologically different^9,10,34^. Body size, or at least body condition, can further be influenced by a species’ diet. Data on natural prey abundance were not collected in either of the two study locations, but provisioning sites in Moorea are numerous, and adult *C. melanopterus* frequent such sites^36^. While direct impacts of provisioning on body condition is either sparsely documented^37,38^ or show minimal impacts on the sharks’ diet^39^, provisioned female *C. melanopterus* may benefit from high-trophic level food, which, in turn, is likely to augment maternal investments (e.g., more endogenous energy resources) for their offspring. Further, the exclusive economic zone (EEZ) of French Polynesia banned fishing for *C. melanopterus* in 2006^40^. This fishing ban may have helped protect larger and better conditioned females, which in turn give birth to larger, heavier, and better conditioned offspring^12^. Lastly, it could be argued that differences in fecundity influence variable pup sizes in Moorea and St. Joseph. While the estimated average litter size of sharks from Moorea and Aldabra (~900 km southwest of St. Joseph) is three pups, Moorea’s sharks demonstrate annual reproductive cycles as opposed to biennial cycles in Seychelles^34,41,42^. This suggests that, considering the very limited data, fecundity does not explain our findings, because more frequent cycles in Moorea would likely infer smaller pups. While any of these mechanisms alone or in combination could explain the intraspecific variation in the level of maternal investment of female *C. melanopterus*, identifying the specific factors that result in female sharks in Moorea being larger and giving birth to larger, heavier, and better conditioned offspring was beyond the scope of this study.

The rate at which body condition and autonomous foraging success changed as umbilical scars began disappearing varied between Moorea and St. Joseph sharks, suggesting early development may be site-specific for young *C. melanopterus*. Although the rapid decrease of body condition in *C. melanopterus* from Moorea is not surprising, considering documented declines in body conditions in other young sharks^12,16,17,22^, the relatively high maternal investment in Moorea was expected to lead to slower declines in body condition (e.g., due to more energy reserves at birth) and faster foraging development and success compared to sharks from St. Joseph. Our study, however, demonstrates significant declines in body condition and slower foraging development in sharks from Moorea, therefore suggesting that the quality of the maternal energy investment is not correlated with the foraging success of the young. Other factors, such as environmental conditions, prey resources, variable foraging strategies, and/or anthropogenic stressors are all likely, in some part, to be responsible for the observed differences across sites.

Environmental conditions, such as seawater temperatures, were measured in Moorea and St. Joseph. Despite significantly lower mean temperatures during pupping seasons in Moorea (29.5 °C ± 0.003) compared to St. Joseph (30.0 °C ± 0.003), temperature ranges were highly comparable (see Supplementary Information S4). These small differences in mean temperatures lead to standard metabolic rates (SMR; the cost of maintenance metabolism) of 160.5 and 162.7 mg O_2_ kg^−1^ h^−1^, respectively (Bouyoucos, IA, unpublished data). A difference in SMR of 1.4% is, however, negligible in maintenance costs and is therefore likely not responsible for the observed site-specific differences in changes of body condition. However, if ocean temperatures continue to increase, a decrease in body condition during early life-stages may be more pronounced, because higher water temperatures can have decelerating effects on growth^43^.

Variable rates of decreasing body condition and foraging development in young sharks may have also been shaped by different levels of inter- and intraspecific competition in young sharks for limited prey resources. Recent studies categorize nearshore areas as resource-limited, a condition that may especially be distinctive in remote areas, where multiple juvenile shark species co-occur and compete for similar prey^44–46^. Both Moorea and St. Joseph are inhabited by multiple populations of young sharks^13,20^, therefore, competition is likely to occur at both locations^18^. Indeed, co-occurrence and potential competition in Moorea lead to isotopic niche partitioning between juvenile *C. melanopterus* and sicklefin lemon sharks (*Negaprion acutidens*); yet, body condition as well as growth rates were not affected by the coexisting species^45^. Even if prey abundances were not quantitatively assessed in any of the two study sites, small reef-associated teleosts (e.g., the predominant prey of young sharks^41,47^) are often observed in St. Joseph at site of collection (Weideli, OC, personal observation) and in 85% of stomachs investigated. These observations suggest that prey availability at St. Joseph is sufficient, resulting in potentially weak competitive interactions between young sharks. At Moorea, during gillnet deployments (n = 175) for this study, potential prey species were rarely observed; although this does not prove their absence. Future studies assessing competitive patterns among coexisting shark species and prey availability are, however, needed to draw further conclusions as to why body condition and foraging development during the first weeks of life change at different rates in Moorea and St. Joseph.

In addition to prey availability, the caloric value of ingested prey as well as foraging strategies may differ between sites. Juvenile scalloped hammerheads (*Sphyrna lewini*) have been reported consuming energetically poor prey^16,48^, which may explain the observed decreases in body mass after parturition^16^. The liver lipids that young sharks in Moorea receive as a maternal headstart are potentially higher in energy compared to their ingested prey. This caloric difference may help explain the body condition decrease as their umbilical scars begin to disappear, similar to the loss of maternal isotopic signals observed in young bull sharks (*Carcharhinus leucas*) and Atlantic sharpnose sharks (*Rhizoprionodon terraenovae*)^22^. On the contrary, small or negligible differences in energetic value of maternal energy resources compared to young sharks’ prey may explain the maintained body condition observed in St. Joseph sharks. Low caloric prey may also help to explain how an increase in foraging success from 30% and 47% to 51% of stomachs containing prey (Fig. 7) can result in decreasing body condition in Moorea sharks. Similar findings have been reported by Hussey *et al*.^12^, where body condition of neonatal dusky sharks (*Carcharhinus obscurus*) decreased despite increasing stomach content mass (increasing feeding activities). Nonetheless, this is highly speculative, and more stomach items, especially those from extremely young sharks (e.g., USS1 fresh umbilical scars), as well as the actual caloric value of the stomach contents are needed to better understand the relationship between decreasing body condition despite increasing foraging success.

Prey resources and their caloric value may deteriorate in nearshore areas with substantial anthropogenic impacts^28,49^. Indeed, the abundance of small reef-associated teleosts is declining through large-scale habitat degradation^27,50^, and artisanal fishing^51^. Likewise, anthropogenic habitat degradation underpins the declines in the abundance of energetically high-value prey species (e.g., small scarids) with a concurrent increase of low caloric gobies and shrimps in the shallow areas of Kāne’ ohe Bay, Hawai’i (USA)^52^. This transition to lower caloric-value prey is thought to be partially responsible for the declining body mass in *S. lewini* during their first weeks of life^16^. Anthropogenic stressors, however, can also have direct impacts on young sharks. Increasing temperatures and salinity, for example, allowed young *C. leucas* to expand into formerly uninhabited bays^53^ with potentially different prey resources and also into areas where artisanal nearshore fisheries frequently capture young sharks^54,55^. Even if young sharks are not the target species in artisanal fisheries and are subsequently released, accidental capture events cause stress^56^. Young *C. melanopterus*, for example, require at least 8 h recovery after a single accidental gillnet capture event; during this time, about 15% of the energy used for daily swimming is lost^56^. Despite enforcement of partially protected areas (no-take zones) around Moorea^57^, artisanal fishing is far more likely to occur within the coastal areas of Moorea when compared to near-pristine and uninhabited St. Joseph, with its uninterrupted reserve boundary^35,40^. Similarly, human activities at Moorea (e.g., boat traffic, boat channel dredging, and shoreline activities) may constrain young shark habitats, with sharks potentially avoiding deeper channels or areas with boat traffic.

The observed relationship between decreasing body condition with increasing USS in Moorea sharks is further supported by data from individual sharks that were captured on multiple occasions. This is, to our best knowledge, the first evidence of a significant decrease of body condition with time at liberty in individual wild sharks (Fig. 5). Results from such recaptures also depict that individuals with higher body condition indices (K as well as GF) at initial capture had more pronounced decreases in body condition during the first weeks of life (Fig. 6). This is analogous to the findings across habitats, in which sharks from Moorea with higher maternal investments were subject to significant decreases in body condition (Fig. 4a,c) compared to sharks from St. Joseph, where such a decline was absent (Fig. 4b,d). Since all recaptured individuals at Moorea were exposed to similar environmental conditions (e.g., prey availability, prey quality, and anthropogenic stressors), other factors must have contributed to the within-population differences around Moorea. One plausible answer could be that sharks with higher initial body condition are less driven to start foraging because they can rely on ample endogenous energy resources for an extended period of time. On the contrary, individuals with lower initial body condition are forced to develop foraging skills at an earlier age, hence demonstrating a positive change in body condition between capture events. This is speculative, because unexperienced young sharks are generally considered as asynchronous opportunistic foragers^58^, and dietary information were not collected from recaptured sharks. Also, body condition is only a proxy that may mask other behavioural or physiological traits that may have influenced our findings. Future work should therefore aim to collect dietary information (e.g., stomach contents or isotopic information) from recaptured sharks to validate changes in body condition between multiple capture events. Finally, prospective studies are recommended to include long-term recaptures to elucidate whether the body condition changes that are observed during early-life stages influence later development stages or if these early body condition changes are negligible for older age-classes.

In conclusion, our findings suggest and support that decreases in body condition within the first weeks of life are common for young viviparous sharks and not only result from natural depletions of maternal energy resources, but will also in some part be affected by prey availability, prey quality, foraging strategies, and/or anthropogenic stressors^12,16^. Our approach, using two populations of *C. melanopterus*, further enabled us to discriminate between different maternal investments in which young sharks from Moorea with higher maternal energy resources were found to demonstrate significant decreases in body condition and slower foraging development compared to sharks from St. Joseph. A comparable observation was provided within the Moorea population in which better-conditioned individuals were subject to a higher loss of body condition. It is therefore expected that young sharks with relatively lower body condition are forced to develop foraging skills at an earlier life-stage, resulting in higher proportions of stomachs containing prey and a positive change in body condition between recaptures. This finding suggests that the habitat quality (e.g., prey abundance and quality) might be especially important for sharks with limited maternal energy resources, and generally for sharks that occur in isolated, nearshore habitats, where deeper surrounding waters or anthropogenically-induced channels impede or prevent dispersal to nearby, potentially prey-rich habitats.

The continued global expansion of human activities (e.g., overfishing, climate change, coastal development, and pollution) poses the greatest risk to reef-associated, shallow water shark species^59^. Therefore generating site-specific information on early development of reef sharks is critical^60^. During these early life-stages, young sharks not only depend on the maternal energy resources, but also rely on these nearshore areas for shelter and/or to access adequate prey resources. Therefore, to achieve sound conservation measures for *C. melanopterus* and other viviparous reef sharks, management strategies need to come together to effectively protect breeding populations as well as young sharks and their shallow nearshore habitats.

## Methods

### Study location and sampling

Some of the sharks for this study were captured as part of long-term fisheries-independent surveys in Moorea, French Polynesia (17°30′S, 149°51′W). Moorea is surrounded by fringing reefs and lagoons that are adjacent to shallow nearshore areas serving as putative nursery grounds for young *C. melanopterus*^34,45^. Juvenile *C. melanopterus* were captured using gillnets (50.0 m × 1.5 m, 5.0 cm mesh) during the parturition months (September – February) in 2016/2017 and 2017/2018. Captured individuals were immediately removed from the net, and handling time was kept to a minimum (<7 min.) to avoid excessive capture-related stress^56^. Sharks captured in 2016/2017 were externally tagged using coloured T-bar anchor tags (Hallprint ®, Hindmarsh Valley, SA, Australia) and internally with passive integrated transponder (PIT) tags (Biolog-ID) in 2017/2018 to allow recaptured animals to be identified. During these sampling events, pre-caudal length (L_PC,_ the length from the tip of the snout to the precaudal notch) and three girth measurements were measured to the nearest 0.1 cm with a tape measure for each shark: 1) pectoral girth (G_PEC_), the circumference of the shark measured at the base of the pectoral fin insertion, anterior to the dorsal fin, 2) dorsal girth (G_DOR_), the circumference measured at the base of the first dorsal fin insertion, and 3) caudal girth (G_CAU_), measured anterior to the caudal fin in the precaudal notch (see Supplementary Information S5). Umbilical scar stage (USS), a reliable indicator of neonatal life-stages^12,16,61^, was quantified into three categories. USS1 was applied if scar was fully open, USS2, if scar was semi-healed, and USS3 for fully healed scars (see Supplementary Information S6). Individuals with USS1 and USS2 were considered as neonate sharks with an estimated maximal age of four weeks^12,16,61^. Sharks with closed scars (USS3) were identified as young-of-the-year (>four weeks old) and no differentiation was made between visible and well-healed scars. We were unable to differentiate between young-of-the-year and older sharks, due to systematic size overlap between different age-classes^11^. The USS of each shark was photographed alongside a ruler, and total body mass (M_TB_) was measured with a hand-held scale to the nearest 10 g. After completing basic measurements, a subset of *C. melanopterus* individuals also had their stomachs flushed, similar to Bangley *et al*.^62^. Different diameters of transparent acrylic tubes (2.5, 3.2, and 3.8 cm outer tube diameter) were used according to the shark sizes (<60 cm, between 60–70 cm and >70 cm L_T_, respectively). The beveled and lubricated tubes were inserted through the mouth, esophagus, and into the stomach while the sharks were kept in the water. As soon as the stomach and the tube were filled with water, the shark was turned upside down to flush the stomach. The stomach items were captured in a sieve, and the percentage of stomachs containing prey was recorded. This procedure was solely conducted on sharks in good condition (e.g., no open wounds) and was kept to a maximum of three consecutive procedures per individual. Environmental temperatures were recorded every ten minutes during parturition season with stationary Hobo® temperature loggers (UA-002-64, Onset Computer Corporation, Bourne, MA, USA) deployed in capture locations.

Fieldwork was further conducted in the western Indian Ocean at St. Joseph atoll (05°26′S, 53°20′E) in the Republic of Seychelles. St. Joseph is a near-pristine and non-inhabited atoll that offers shallow, protected areas for at least two species of young sharks^13^. Juvenile *C. melanopterus* were captured with gillnets (20.0 m × 1.5 m, 5.0 cm mesh) during the parturition months (October – December and March – April) in 2014/2015, 2015/2016 and 2016/2017. Captured *C. melanopterus* were immediately removed from the net, and handling time was kept to a minimum (<7 min.) to avoid excessive capture-related stress^56^. Sharks were internally tagged using PIT tags (Biomark®) to allow recaptured sharks to be identified. The L_PC_, girth, USS, and M_TB_ were measured for each shark, and gastric lavage was subsequently conducted using a sub-sample of sharks following Moorea’s protocol. All sharks were released at site within minutes of capture. Temperatures were recorded every fifteen minutes during parturition season with stationary Hobo® temperature loggers (U22-001, Onset Computer Corporation, Bourne, MA, USA) distributed across the area surveyed.

### Data analyses

Where applicable, data were checked for normality using Shapiro-Wilk tests prior to analyses in R version 3.5.3^63^ within the RStudio interface ver. 1.0.153^64^. For all tests, the level of statistical significance α was set at 0.05, and results are reported as means ± SD. To investigate potential intraspecific life-history variabilities in neonates and temperature differences across habitats, mean L_PC_, M_TB_ and water temperatues were compared with two sample *t*-tests, and frequency histograms were subsequently constructed. Total body mass for a given L_PC_ was used to estimate body condition, assuming that individuals in a good condition would be heavier than those in poorer condition of the same length. Thus, we determined allometric length–mass relationships by using the formula log y = log a + b log x. These coefficients were used in M_TB_ = a L_PC_^b^, where M_TB_ is total body mass (g) and L_PC_ is length (cm).

Two independent indices of individual body condition were also calculated. The Fulton’s body condition index, also known as Fulton’s K^65^, calculates a morphometric index of a fish’s body condition with the following equation:

1 $${\rm{K}}={{\rm{10}}}^{{\rm{5}}}\,{{\rm{M}}}_{{\rm{TB}}}{({{{\rm{L}}}_{{\rm{PC}}}}^{{\rm{3}}})}^{-{\rm{1}}}$$

We also constructed a non-lethal and morphometric condition index, based on the assumption that individuals with larger livers for a given body length are in better condition^12^. Similar to Irschick & Hammerschlag^66^, three measurements along the shark’s body were chosen to incorporate the size and anatomical location of the liver, as well as the shark’s shape, which is wider along the anterior part of the body^67^. While massive body sizes prevent measuring the circumference in previous studies^66,67^, we were able to take three circumference measurements to calculate the girth factor (GF) as a proxy for body condition using the following equation:

2 $${\rm{GF}}=[{{\rm{G}}}_{{\rm{PEC}}}+{{\rm{G}}}_{{\rm{DOR}}}+{{\rm{G}}}_{{\rm{CAU}}}]\,{{{\rm{L}}}_{{\rm{PC}}}}^{-{\rm{1}}}$$

Resulting condition indices (K and GF) were compared across locations using a two sample *t*-tests. To demonstrate and validate the absence of inadvertent co-linearity between the two body condition indices and L_PC_^2,3^, linear least-squares regressions were performed for L_PC_ with K and GF, respectively.

In order to follow the transition of body condition with the closure of the umbilicus (increasing USS), analysis of variance (ANOVA) and Tukey’s honest significant difference (HSD) tests were used for post-hoc multiple comparisons for Fulton’s K at Moorea. As the other comparisons did not conform to a normal distribution, Kruskal-Wallis tests were applied. Post-hoc multiple comparisons were subsequently evaluated using Dunn tests, while p-values were adjusted using the Holm method to reduce type I error. In addition, individual sharks from Moorea that were captured on multiple occasions were used to further validate body condition changes during early life stages. Recaptured individuals allowed us to calculate the change in body condition between two capture events by subtracting body condition indices (K and GF) of the initial capture from the recapture event. Similarly, recaptured individuals allowed us to estimate if changes in body condition depend on the body condition at initial capture. For both calculations, values were plotted for each individual in a linear least-square regression. Furthermore, Fulton’s K was linearly regressed against girth factor GF of recaptured *C. melanopterus* to demonstrate that changes in K could be predicted by changes in GF. Finally, in order to estimate level of autonomous foraging success during increasing USS, a sub-sample of *C. melanopterus* from Moorea and St. Joseph had gastric lavages performed. The obtained stomach status (% of stomachs containing prey) were compared with Χ^2^ test.

### Ethical approval

Sharks for this study were captured as part of long-term fisheries-independent surveys in Moorea, French Polynesia and on St. Joseph, Republic of Seychelles. Ethical approval for Moorea was given by James Cook University Animal Ethics Committee protocol A2089 and permission to work with sharks in French Polynesia was obtained from the Ministère de l’Environnement (Arrete N° 9524). Research on sharks at St. Joseph was approved by, and conducted with the knowledge of Ministry of Environment, Energy, and Climate Change, Seychelles. Animal handling and tagging methods were conducted in accordance with the approved guidelines of S. Planes by the Autorisation de pratiquer des expériences sur les animaux n° 006725 (1995) from the ministry of Agriculture.

## Supplementary information

Supplementary information
