# Supplementary material for: Same species, different prerequisites: investigating body condition and foraging success in young reef sharks between an atoll and an island system
Source: Sci Rep. 2019 Sep 17;9:13447. doi: 10.1038/s41598-019-49761-2 (PMC6748967; doi:10.1038/s41598-019-49761-2)
Supplement: Supplementary file 1 — Supplementary information [file 41598_2019_49761_MOESM1_ESM.pdf]

Supplementary Information

Same species, different prerequisites: investigating body condition and foraging success in young reef sharks between an atoll and an island system

Ornella C. Weideli*, Ian A. Bouyoucos, Yannis P. Papastamatiou, Gauthier Mescam, Jodie L. Rummer, Serge Planes

*Corresponding author

Email: ornella.weideli@gmail.com

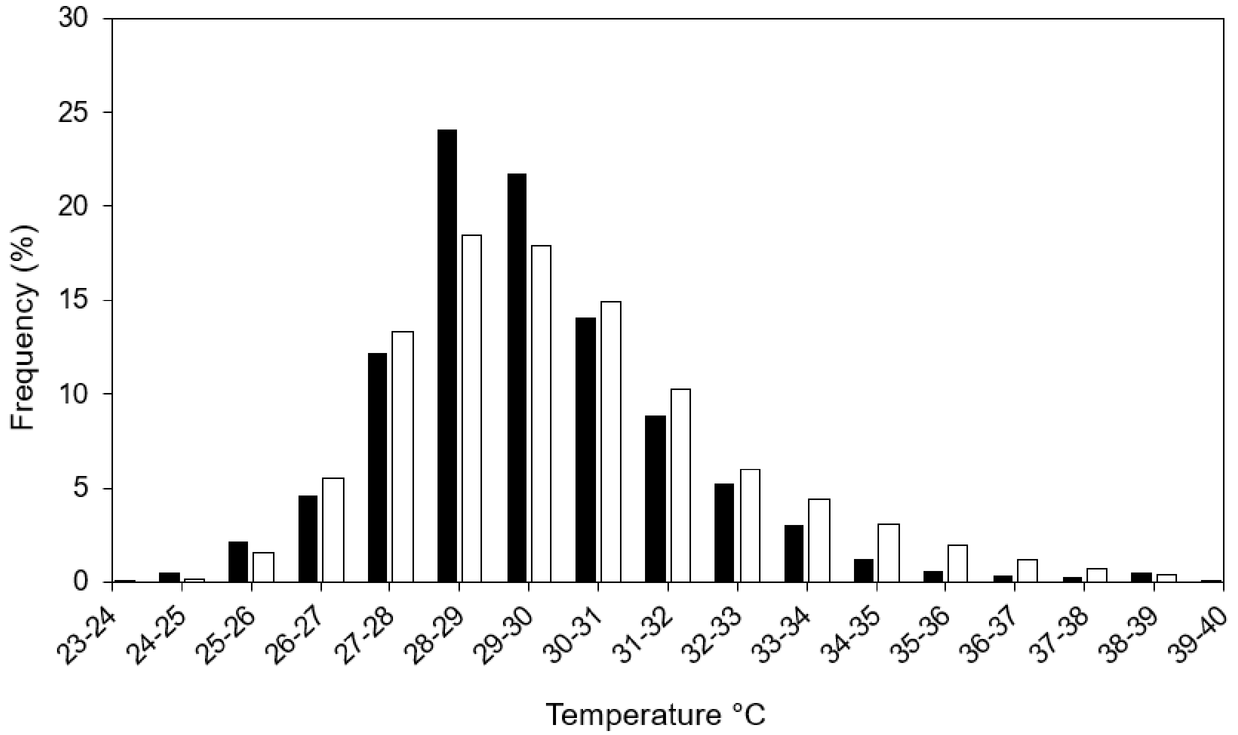

Supplementary Information S1: Percentage frequency histogram of temperature measured during pupping seasons in Moorea (2016/2017 and 2017/2018, black) and St. Joseph (2015/2016, 2016/2017, and 2017/2018, white). Temperature was measured every fifteen and ten minutes in Moorea and St. Joseph, respectively.

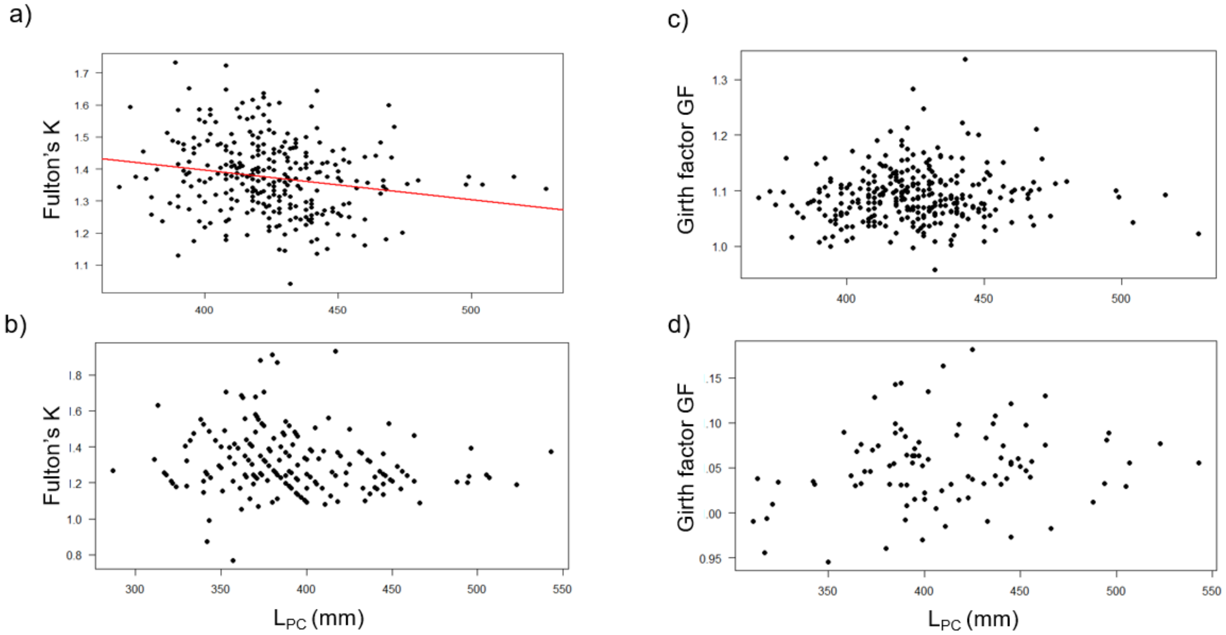

Supplementary Information S2: Scatterplots of pre-caudal length (L_{PC}) and Fulton's K for young *Carcharhinus melanopterus* from a) Moorea (n = 313), and b) St. Joseph (n = 91) and girth factor GF for *Carcharhinus melanopterus* from c) Moorea (n = 313), and d) St. Joseph (n = 224). The regression line for predicting changes in Fulton's K with pre-caudal length (L_{PC}) in Moorea is shown in red ($y = 1.77 – 0.0009x$, $r^2 = 0.04$).

ID	Sex	Initial capture	Recapture	Time (days)	Δ GF	Δ K
cm66	Male	15-Nov-16	28-Nov-16	13	0.033	-0.056
cm68	Female	16-Nov-16	01-Dec-16	15	-0.047	-0.077
cm79	Male	18-Nov-16	02-Dec-16	14	-0.077	-0.052
cm77	Male	18-Nov-16	02-Dec-16	14	-0.043	-0.017
cm85	Male	20-Nov-16	04-Dec-16	14	-0.017	0.050
cm45	Female	02-Nov-16	04-Dec-16	32	-0.006	-0.093
cm71	Male	17-Nov-16	09-Dec-16	22	-0.025	-0.159
cm111	Male	01-Dec-16	10-Dec-16	9	-0.084	-0.144
cm110	Female	01-Dec-16	10-Dec-16	9	-0.070	-0.059
cm112	Female	01-Dec-16	10-Dec-16	9	-0.009	0.041
cm72	Female	17-Nov-16	23-Dec-16	36	-0.060	-0.249
cm112	Female	10-Dec-16	24-Dec-16	14	0.021	-0.146
cm145	Female	13-Dec-16	03-Jan-17	21	-0.050	-0.176
cm158	Female	22-Dec-16	07-Jan-17	16	0.067	0.099
cm155	Female	22-Dec-16	07-Jan-17	16	0.031	0.209
cm159	Male	22-Dec-16	07-Jan-17	16	0.021	0.102
cm173	Male	07-Jan-17	21-Jan-17	14	-0.057	-0.130
cm160	Female	22-Dec-16	21-Jan-17	30	-0.041	-0.041
cm152	Male	19-Dec-16	27-Jan-17	39	-0.011	0.018
cm192	Male	08-Feb-17	20-Feb-17	12	0.005	-0.014
cm192	Male	20-Feb-17	24-Feb-17	4	0.006	0.009
cm190	Male	08-Feb-17	28-Mar-17	48	-0.050	0.161
Cm2	Female	26-Sep-17	08-Oct-17	12	-0.135	-0.124
Cm31	Female	26-Oct-17	13-Nov-17	18	-0.011	-0.058
Cm26	Male	26-Oct-17	13-Nov-17	18	-0.015	-0.109
Cm53	Male	02-Nov-17	14-Nov-17	12	-0.022	0.014
Cm41	Male	30-Oct-17	20-Nov-17	21	-0.050	-0.068
Cm65	Female	07-Nov-17	23-Nov-17	16	-0.039	-0.213
Cm46	Male	02-Nov-17	27-Nov-17	25	-0.001	-0.153
Cm61	Male	06-Nov-17	06-Dec-17	30	-0.055	-0.137
Cm62	Male	06-Nov-17	07-Dec-17	31	-0.070	-0.128
Cm58	Female	06-Nov-17	07-Dec-17	31	-0.100	-0.210
Cm43	Male	31-Oct-17	18-Dec-17	48	0.023	-0.141
Cm102	Male	06-Dec-17	22-Dec-17	16	-0.006	0.055
Cm106	Male	06-Dec-17	22-Dec-17	16	0.036	0.098
Cm101	Female	06-Dec-17	22-Dec-17	16	0.116	-0.004
Cm107	Female	06-Dec-17	22-Dec-17	16	0.037	-0.093
Cm57	Female	06-Nov-17	28-Dec-17	52	-0.088	-0.237
Cm116	Male	18-Dec-17	29-Dec-17	11	0.053	0.110
Cm41	Male	30-Oct-17	10-Jan-18	72	-0.039	-0.207
Cm116	Male	29-Dec-17	15-Jan-18	17	-0.077	-0.129
Cm112	Male	08-Dec-17	16-Jan-18	39	-0.054	-0.051
Cm99	Female	06-Dec-17	17-Jan-18	42	-0.058	-0.045

Supplementary Information S3: Overview of 45 neonatal *Carcharhinus melanopterus* from Moorea that were recaptured within the same parturition season (minimum 4 and maximum 72 days). Values demonstrate the difference (Δ) in body condition indices between initial capture and recapture. GF: Girth factor; K: Fulton's K.

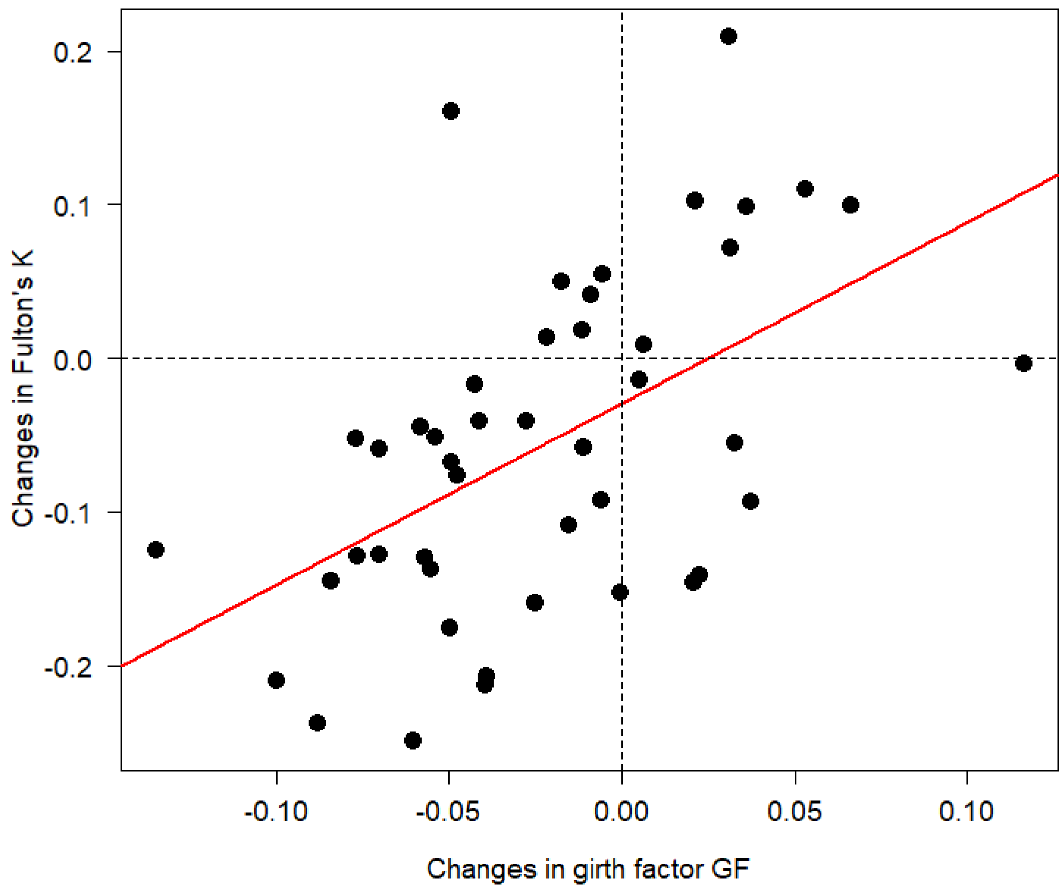

Supplementary Information S4: Changes in Fulton's K versus changes in girth factor GF in neonatal *Carcharhinus melanopterus* from Moorea. Data were obtained from 45 sharks that were measured twice within the same parturition season (minimum 4 days, maximum 72 days). The regression line for predicting changes in Fulton's K from changes in GF is shown in red ($y = -0.03 + 1.18x$, $r^2 = 0.28$).

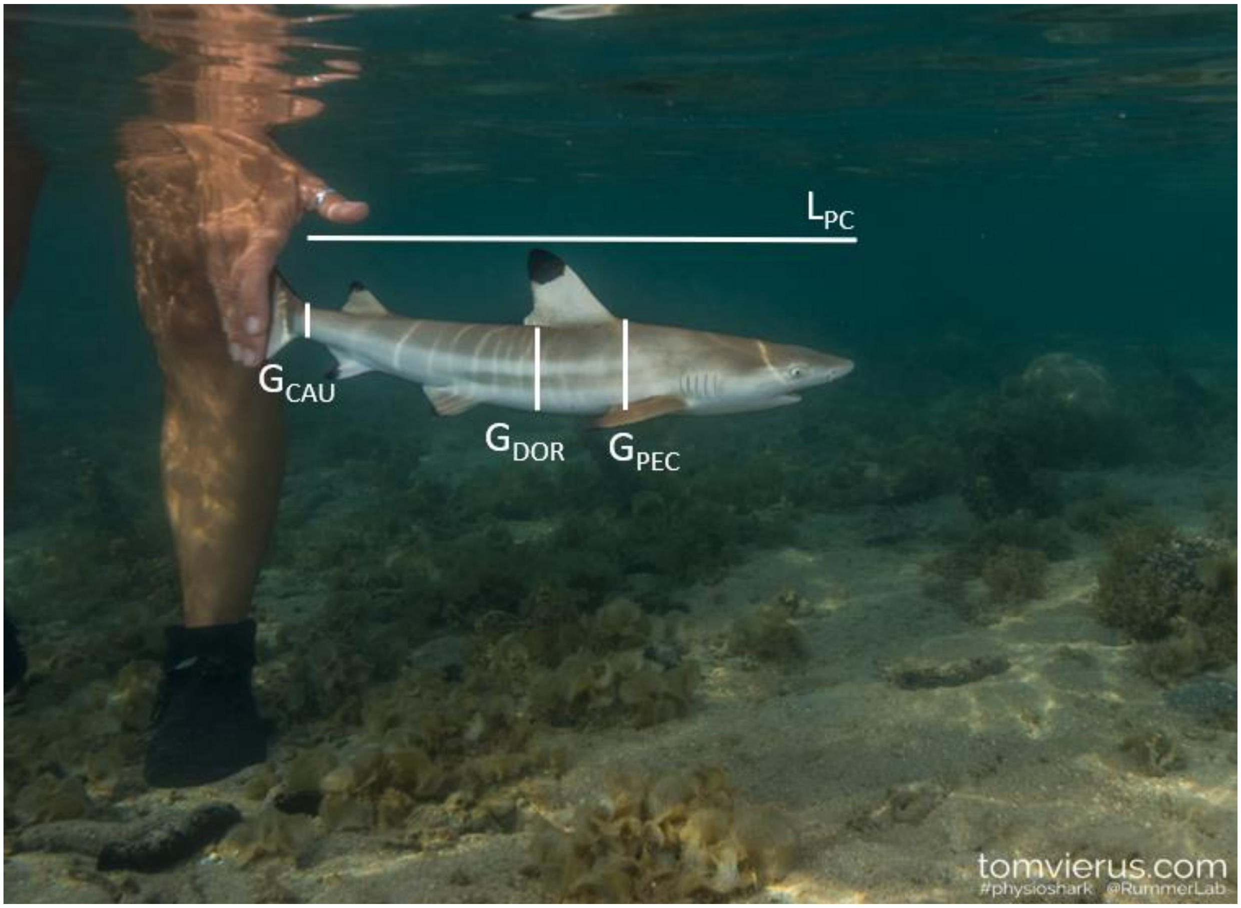

Supplementary Information S5: Location of measurements on juvenile *Carcharhinus melanopterus*: pectoral girth (G_{PEC}), dorsal girth (G_{DOR}), caudal girth (G_{CAU}), and precaudal length (L_{PC}). Picture: Tom Vierus.

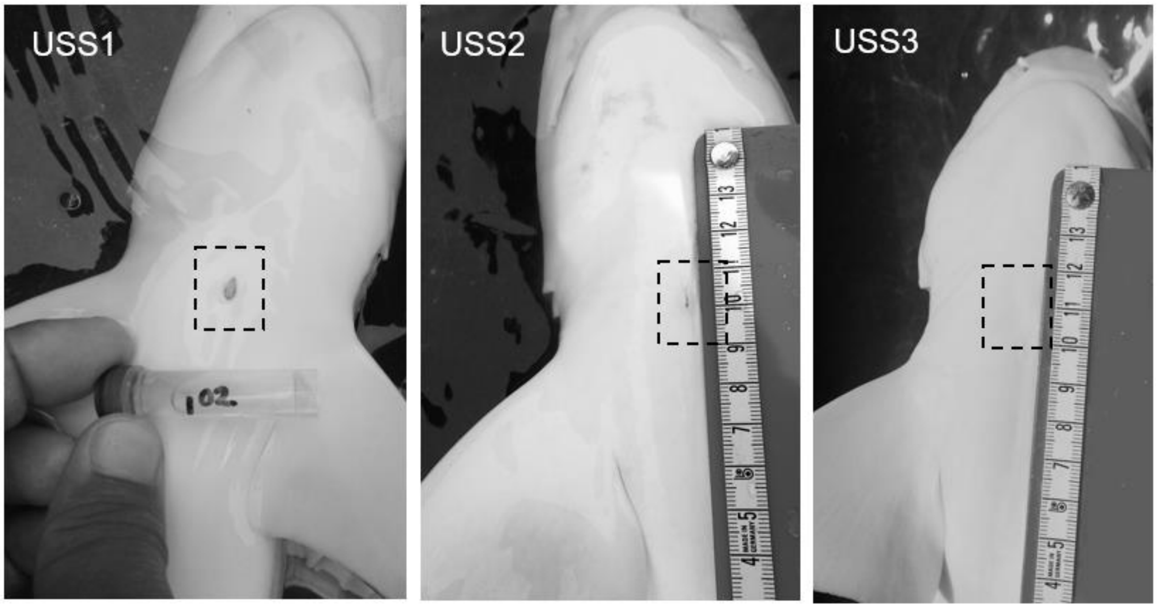

Supplementary Information S6: Umbilical scar stages (USS) in young *Carcharhinus melanopterus*. USS1 = open umbilical scar, USS2 = semi-healed scar, USS3 = fully healed scar. Note that no differentiation is made between visible and well-healed scars in USS3. Picture: Ornella Céline Weideli | SOSF.
